# Tris(ethane-1,2-diamine-κ^2^ *N*,*N*′)cobalt(II) *cis*-aqua-2κ*O*-μ-cyanido-1:2κ^2^ *C*:*N*-hepta­cyanido-1κ^7^ *C*-bis­(ethane-1,2-diamine-2κ^2^ *N*,*N*′)cobalt(II)molybdenum(IV) dihydrate

**DOI:** 10.1107/S1600536808033564

**Published:** 2008-10-18

**Authors:** W. Purcell, Hendrik G. Visser

**Affiliations:** a Department of Chemistry, University of the Free State, PO Box 339, Bloemfontein 9300, South Africa

## Abstract

The title compound, [Co(C_2_H_8_N_2_)_3_][CoMo(CN)_8_(C_2_H_8_N_2_)_2_(H_2_O)]·2H_2_O, is isostructural with the Ni^II^ analogue. The Mo^IV^ atom is coordinated by eight cyanide ligands, one of which forms a bridge to a Co^II^ atom that is itself coordinated by two bidentate ethane-1,2-diamine (en) ligands and one water mol­ecule. Another Co^II^ complex, coordinated to three bidentate en ligands, acts as the counter-ion. The crystal structure contains O—H⋯N/O, N—H⋯N/O and C—H⋯N/O hydrogen bonds, which form a three-dimensional network.

## Related literature

For the isostructural Ni^II^ compound, see: Withers *et al.* (2005 ▶); Chang *et al.* (2002 ▶). For other similar complexes and syntheses, see: Przychodzen *et al.* (2006 ▶); Holmes *et al.* (2002 ▶); Beauvais & Long (2001 ▶); Leipoldt *et al.* (1974 ▶).
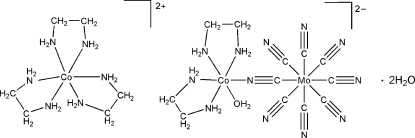


## Experimental

### 

#### Crystal data

[Co(C_2_H_8_N_2_)_3_][CoMo(CN)_8_(C_2_H_8_N_2_)_2_(H_2_O)]·2H_2_O
*M* *_r_* = 776.53
Orthorhombic, 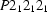

*a* = 11.5377 (3) Å
*b* = 14.8830 (3) Å
*c* = 18.7376 (4) Å
*V* = 3217.54 (13) Å^3^
*Z* = 4
Mo *K*α radiation
μ = 1.45 mm^−1^
*T* = 100 (2) K
0.35 × 0.26 × 0.20 mm

#### Data collection

Bruker APEXII CCD diffractometer
Absorption correction: multi-scan (*SADABS*; Bruker, 2004 ▶) *T*_min_ = 0.640, *T*_max_ = 0.750
41911 measured reflections
8019 independent reflections
6732 reflections with *I* > 2σ(*I*)
*R*_int_ = 0.077

#### Refinement

*R*[*F* ^2^ > 2σ(*F* ^2^)] = 0.040
*wR*(*F* ^2^) = 0.093
*S* = 1.06
8019 reflections
397 parameters
7 restraints
H atoms treated by a mixture of independent and constrained refinement
Δρ_max_ = 0.46 e Å^−3^
Δρ_min_ = −1.64 e Å^−3^
Absolute structure: Flack (1983 ▶), 3559 Friedel pairs
Flack parameter: −0.063 (15)

### 

Data collection: *APEX2* (Bruker, 2005 ▶); cell refinement: *SAINT-Plus* (Bruker, 2004 ▶); data reduction: *SAINT-Plus* and *XPREP* (Bruker, 2004 ▶); program(s) used to solve structure: *SHELXS97* (Sheldrick, 2008 ▶); program(s) used to refine structure: *SHELXL97* (Sheldrick, 2008 ▶); molecular graphics: *DIAMOND* (Brandenburg & Putz, 2005 ▶); software used to prepare material for publication: *SHELXL97*.

## Supplementary Material

Crystal structure: contains datablocks global, I. DOI: 10.1107/S1600536808033564/bi2302sup1.cif

Structure factors: contains datablocks I. DOI: 10.1107/S1600536808033564/bi2302Isup2.hkl

Additional supplementary materials:  crystallographic information; 3D view; checkCIF report

## Figures and Tables

**Table 1:** Selected bond lengths (Å)

Mo1—C7	2.149 (4)
Mo1—C1	2.149 (4)
Mo1—C2	2.150 (4)
Mo1—C6	2.156 (4)
Mo1—C3	2.161 (4)
Mo1—C5	2.164 (4)
Mo1—C8	2.168 (4)
Mo1—C4	2.169 (4)
Co2—N27	2.065 (3)
Co2—N36	2.099 (3)
Co2—N37	2.109 (3)
Co2—N35	2.109 (3)
Co2—N38	2.113 (3)
Co2—O41	2.118 (3)
Co1—N32	2.106 (4)
Co1—N30	2.122 (3)
Co1—N34	2.126 (3)
Co1—N33	2.130 (3)
Co1—N29	2.131 (3)
Co1—N31	2.132 (3)

**Table 2:** Hydrogen-bond geometry (Å, °)

*D*—H⋯*A*	*D*—H	H⋯*A*	*D*⋯*A*	*D*—H⋯*A*
N29—H29*A*⋯N22^i^	0.90	2.59	3.313 (5)	138
N29—H29*B*⋯N24^ii^	0.90	2.45	3.243 (5)	147
N30—H30*A*⋯N26^iii^	0.90	2.39	3.121 (5)	139
N31—H31*B*⋯N24^ii^	0.90	2.22	3.079 (4)	160
N32—H32*A*⋯N22^i^	0.90	2.4	3.163 (5)	143
N32—H32*B*⋯N26^iv^	0.90	2.16	3.037 (5)	164
N33—H33*B*⋯O41^v^	0.90	2.51	3.233 (4)	138
N34—H34*A*⋯N28^iv^	0.90	2.53	3.213 (5)	133
N34—H34*B*⋯N26^iii^	0.90	2.53	3.408 (5)	164
N36—H36*A*⋯N22^vi^	0.90	2.31	3.195 (4)	166
N36—H36*B*⋯N23^vi^	0.90	2.56	3.151 (5)	124
N37—H37*A*⋯N22^vi^	0.90	2.22	3.103 (5)	167
N37—H37*B*⋯N21^vii^	0.90	2.17	3.035 (5)	162
N38—H38*A*⋯N25^viii^	0.90	2.49	3.282 (5)	148
N38—H38*B*⋯O42^vi^	0.90	2.47	3.350 (5)	164
O41—H41*A*⋯N25^viii^	0.84 (2)	1.94 (2)	2.764 (4)	170 (4)
O41—H41*B*⋯O43^vi^	0.85 (2)	1.89 (2)	2.724 (4)	168 (4)
O42—H42*A*⋯N28^v^	0.82 (4)	2.10 (4)	2.924 (4)	173 (4)
O42—H42*B*⋯N23	0.84 (4)	1.96 (4)	2.799 (4)	174 (4)
O43—H43*A*⋯N24^v^	0.85 (2)	2.15 (2)	2.995 (4)	174 (4)
O43—H43*B*⋯O42^ix^	0.84 (3)	1.95 (2)	2.778 (4)	168 (4)
C10—H10*B*⋯N23^iii^	0.97	2.53	3.328 (5)	139
C11—H11*B*⋯O42^ix^	0.97	2.59	3.430 (5)	145

Symmetry codes: (i) 
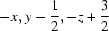
; (ii) 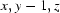; (iii) 
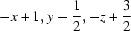
; (iv) 
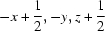
; (v) 
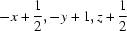
; (vi) 
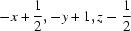
; (vii) 
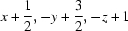
; (viii) 
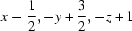
; (ix) 
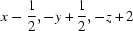
.

**Fig. 1.**
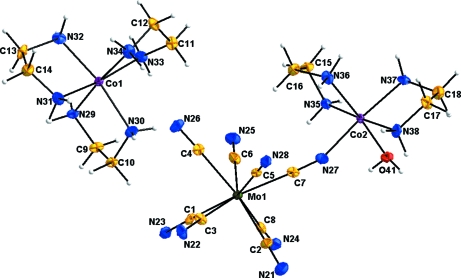
Molecular structure of the title compound with displacement ellipsoids shown at 50% probability for non-H atoms. The lattice water molecules are omitted.

## supplementary crystallographic information

### Comment

The use of cyanometalates as molecular building blocks for potentially constructing clusters and networks with adjustable magnetic properties has developed a lot of interest over the last few years (Beauvais & Long, 2001; Przychodzen *et al.*, 2006; Withers *et al.*, 2005).

The title compound, [Co(en)_3_][Co(H_2_O)(en)_2_(µ_2_-NC)Mo(CN)_7_].2H_2_O (en = 1,2 diaminoethane), is isostructural with its Ni^II^ analogue (Withers *et al.*, 2005; Chang *et al.*, 2002). The Mo^IV^ metal centre is coordinated by eight cyanide ligands, one of which forms a bridge towards a Co^II^ metal centre that is itself coordinated by two bidentate en ligands and a water molecule (Fig. 1). Another Co^II^ complex, coordinated to three bidentate en ligands, acts as counter ion. The octahedral geometry around the two Co^II^ atoms is slightly distorted, as illustrated by the bite angles of the bidentate en ligands, which vary between 81.55 (12) and 94.55 (13) °. The eight-coordinate Mo^IV^ atom forms a slightly distorted square antiprism with the cyanide ligands.

The crystal structure shows a range of hydrogen-bonding of the types N—H···N/O, O—H···N/O and C—H···N/O (Table 2), forming a three-dimensional network.

### Experimental

The synthesis of K_4_[Mo(CN)_8_] and [Co(en)_3_]^2+^ is described elsewhere (Leipoldt *et al.*, 1974; Holmes *et al.*, 2002). The title compound was prepared by adding aqueous soloutions of [Mo(CN)_8_]^4-^ and [Co(en)_3_]^2+^ (1: 2 mol ratio) and allowing to stand for several days. Red plate-like crystals were obtained after several days. The presence of cobalt was confirmed by Inductively Coupled Plasma (IPC) analysis.

### Refinement

The H atoms of the water molecules were located in a difference Fourier map and their positional parameters refined with *U*_iso_(H) = 1.5*U*_eq_(O), and with the O—H distances restrained to be 0.84 (1) Å. Other H atoms were placed geometrically and allowed to ride during subsequent refinement.

### Crystal data

**Table:**

[Co(C_2_H_8_N_2_)_3_][CoMo(CN)_8_(C_2_H_8_N_2_)_2_(H_2_O)]·2H_2_O	*F*(000) = 1600
*M**_r_* = 776.53	*D*_x_ = 1.603 Mg m^−^^3^
Orthorhombic, *P*2_1_2_1_2_1_	Mo *K*α radiation, λ = 0.71073 Å
Hall symbol: P 2ac 2ab	Cell parameters from 5696 reflections
*a* = 11.5377 (3) Å	θ = 2.1–28.3°
*b* = 14.8830 (3) Å	µ = 1.45 mm^−^^1^
*c* = 18.7376 (4) Å	*T* = 100 K
*V* = 3217.54 (13) Å^3^	Plate, red
*Z* = 4	0.35 × 0.26 × 0.20 mm

### Data collection

**Table:**

Bruker APEXII CCD diffractometer	6732 reflections with *I* > 2σ(*I*)
φ and ω scans	*R*_int_ = 0.077
Absorption correction: multi-scan (SADABS; Bruker, 2004)	θ_max_ = 28.3°, θ_min_ = 2.1°
*T*_min_ = 0.640, *T*_max_ = 0.750	*h* = −15→15
41911 measured reflections	*k* = −19→19
8019 independent reflections	*l* = −24→24

### Refinement

**Table:**

Refinement on *F*^2^	H atoms treated by a mixture of independent and constrained refinement
Least-squares matrix: full	*w* = 1/[σ^2^(*F*_o_^2^) + (0.0424*P*)^2^] where *P* = (*F*_o_^2^ + 2*F*_c_^2^)/3
*R*[*F*^2^ > 2σ(*F*^2^)] = 0.040	(Δ/σ)_max_ = 0.028
*wR*(*F*^2^) = 0.093	Δρ_max_ = 0.46 e Å^−^^3^
*S* = 1.06	Δρ_min_ = −1.64 e Å^−^^3^
8019 reflections	Absolute structure: Flack (1983), 3559 Friedel pairs
397 parameters	Flack parameter: −0.063 (15)
7 restraints	

### Special details

**Table:**

Geometry. All e.s.d.'s (except the e.s.d. in the dihedral angle between two l.s. planes) are estimated using the full covariance matrix. The cell e.s.d.'s are taken into account individually in the estimation of e.s.d.'s in distances, angles and torsion angles; correlations between e.s.d.'s in cell parameters are only used when they are defined by crystal symmetry. An approximate (isotropic) treatment of cell e.s.d.'s is used for estimating e.s.d.'s involving l.s. planes.
Refinement. Refinement of *F*^2^ against ALL reflections. The weighted *R*-factor *wR* and goodness of fit *S* are based on *F*^2^, conventional *R*-factors *R* are based on *F*, with *F* set to zero for negative *F*^2^. The threshold expression of *F*^2^ > σ(*F*\2\) is used only for calculating *R*-factors(gt) *etc*. and is not relevant to the choice of reflections for refinement. *R*-factors based on *F*^2^ are statistically about twice as large as those based on *F*, and *R*- factors based on ALL data will be even larger.

### Fractional atomic coordinates and isotropic or equivalent isotropic displacement parameters (Å^2^)

**Table:**

	*x*	*y*	*z*	*U*_iso_*/*U*_eq_	
Mo1	0.24254 (2)	0.51274 (2)	0.608619 (15)	0.00886 (7)	
Co2	0.25983 (4)	0.73223 (3)	0.38903 (2)	0.00726 (10)	
Co1	0.22748 (4)	−0.17502 (3)	0.89163 (2)	0.00804 (10)	
N33	0.1756 (3)	−0.0605 (2)	0.95186 (16)	0.0147 (7)	
H33B	0.1844	−0.0102	0.9258	0.018*	
H33A	0.1006	−0.0653	0.9645	0.018*	
N29	0.1544 (3)	−0.1322 (2)	0.79287 (16)	0.0143 (7)	
H29B	0.1481	−0.1792	0.7629	0.017*	
H29A	0.0831	−0.1094	0.8002	0.017*	
N34	0.3084 (3)	−0.2088 (2)	0.99000 (17)	0.0173 (8)	
H34A	0.2952	−0.2669	1.0005	0.021*	
H34B	0.3855	−0.2003	0.9868	0.021*	
N30	0.3729 (3)	−0.1043 (2)	0.85102 (16)	0.0161 (7)	
H30B	0.3806	−0.0513	0.8738	0.019*	
H30A	0.4379	−0.1365	0.858	0.019*	
N31	0.2809 (3)	−0.3007 (2)	0.84807 (17)	0.0156 (7)	
H31B	0.2915	−0.2953	0.8007	0.019*	
H31A	0.3486	−0.3176	0.8678	0.019*	
N32	0.0747 (3)	−0.2480 (2)	0.91233 (17)	0.0166 (8)	
H32B	0.0739	−0.267	0.9579	0.02*	
H32A	0.0121	−0.213	0.905	0.02*	
C9	0.2306 (3)	−0.0629 (3)	0.76151 (19)	0.0168 (8)	
H9B	0.215	−0.0051	0.7834	0.02*	
H9A	0.2159	−0.0578	0.7107	0.02*	
C11	0.2503 (4)	−0.0570 (2)	1.01647 (18)	0.0171 (8)	
H11B	0.2164	−0.0173	1.0518	0.02*	
H11A	0.3265	−0.0343	1.0041	0.02*	
C12	0.2599 (4)	−0.1509 (2)	1.04599 (18)	0.0167 (8)	
H12B	0.3099	−0.1512	1.0876	0.02*	
H12A	0.184	−0.1726	1.0601	0.02*	
C13	0.0728 (4)	−0.3255 (3)	0.8632 (2)	0.0175 (9)	
H13A	0.0527	−0.3056	0.8155	0.021*	
H13B	0.015	−0.3686	0.8788	0.021*	
C14	0.1913 (3)	−0.3694 (3)	0.8625 (2)	0.0167 (9)	
H14B	0.2061	−0.3976	0.9083	0.02*	
H14A	0.1938	−0.4155	0.826	0.02*	
C10	0.3545 (3)	−0.0890 (3)	0.7740 (2)	0.0167 (9)	
H10A	0.3724	−0.1434	0.7477	0.02*	
H10B	0.4055	−0.0417	0.7574	0.02*	
C1	0.3326 (3)	0.4932 (3)	0.70783 (19)	0.0135 (8)	
C6	0.4102 (3)	0.5675 (3)	0.58215 (19)	0.0146 (8)	
N23	0.3798 (3)	0.4855 (2)	0.76167 (17)	0.0179 (7)	
N25	0.4996 (3)	0.5958 (2)	0.56850 (18)	0.0209 (8)	
C7	0.2162 (3)	0.6069 (3)	0.52324 (19)	0.0115 (8)	
C2	0.0689 (3)	0.5478 (3)	0.63968 (19)	0.0138 (8)	
N27	0.2112 (3)	0.6594 (2)	0.47794 (16)	0.0164 (7)	
N22	0.1263 (3)	0.3320 (2)	0.68324 (17)	0.0182 (7)	
N21	−0.0255 (3)	0.5631 (2)	0.65610 (18)	0.0194 (8)	
C3	0.1686 (3)	0.3946 (3)	0.65782 (19)	0.0126 (8)	
C5	0.1426 (3)	0.4461 (3)	0.52647 (18)	0.0115 (8)	
C8	0.2476 (4)	0.6432 (2)	0.65994 (17)	0.0130 (7)	
C4	0.3543 (3)	0.4051 (3)	0.57178 (19)	0.0139 (8)	
O42	0.5286 (3)	0.4769 (2)	0.87944 (16)	0.0277 (8)	
O43	0.1617 (3)	0.1648 (2)	1.06962 (15)	0.0238 (7)	
N24	0.2504 (3)	0.7142 (2)	0.68527 (16)	0.0187 (7)	
N26	0.4141 (3)	0.3472 (2)	0.55381 (18)	0.0200 (8)	
N28	0.0874 (3)	0.4089 (2)	0.48443 (16)	0.0153 (7)	
N35	0.4319 (3)	0.7425 (2)	0.42627 (17)	0.0137 (7)	
H35A	0.4587	0.7985	0.4193	0.016*	
H35B	0.4346	0.7304	0.4733	0.016*	
N36	0.3217 (3)	0.6119 (2)	0.34467 (17)	0.0170 (7)	
H36B	0.2708	0.5672	0.3526	0.02*	
H36A	0.3309	0.6182	0.2972	0.02*	
C15	0.5036 (3)	0.6773 (3)	0.3866 (2)	0.0197 (8)	
H15B	0.5751	0.6659	0.4123	0.024*	
H15A	0.523	0.7012	0.34	0.024*	
C16	0.4346 (4)	0.5905 (3)	0.3787 (2)	0.0192 (9)	
H16A	0.4773	0.5481	0.3494	0.023*	
H16B	0.4219	0.5635	0.4252	0.023*	
O41	0.2305 (2)	0.85628 (18)	0.44139 (13)	0.0145 (6)	
N37	0.2881 (3)	0.8069 (2)	0.29496 (16)	0.0138 (7)	
H37B	0.3342	0.8543	0.3041	0.017*	
H37A	0.3226	0.7724	0.2617	0.017*	
N38	0.0902 (3)	0.7244 (2)	0.34692 (17)	0.0154 (7)	
H38B	0.0657	0.667	0.3475	0.019*	
H38A	0.0415	0.7573	0.3738	0.019*	
C17	0.0904 (3)	0.7585 (3)	0.2730 (2)	0.0171 (9)	
H17A	0.013	0.7774	0.2595	0.02*	
H17B	0.1151	0.7115	0.2404	0.02*	
C18	0.1737 (3)	0.8378 (3)	0.2691 (2)	0.0166 (9)	
H18A	0.18	0.859	0.2203	0.02*	
H18B	0.1457	0.8868	0.2986	0.02*	
H41A	0.1590 (17)	0.866 (3)	0.442 (2)	0.022*	
H41B	0.259 (3)	0.856 (3)	0.4833 (13)	0.022*	
H42A	0.491 (4)	0.507 (3)	0.9084 (19)	0.042*	
H42B	0.488 (4)	0.481 (3)	0.8422 (19)	0.042*	
H43A	0.189 (3)	0.201 (2)	1.0999 (17)	0.036*	
H43B	0.119 (3)	0.127 (2)	1.0899 (19)	0.036*	

### Atomic displacement parameters (Å^2^)

**Table:**

	*U*^11^	*U*^22^	*U*^33^	*U*^12^	*U*^13^	*U*^23^
Mo1	0.00841 (14)	0.00942 (14)	0.00875 (14)	−0.00039 (12)	0.00007 (14)	−0.00012 (11)
Co2	0.0074 (2)	0.0068 (2)	0.0076 (2)	−0.00054 (18)	−0.0008 (2)	0.00084 (18)
Co1	0.0077 (2)	0.0081 (2)	0.0083 (2)	0.00020 (18)	−0.0002 (2)	−0.00015 (19)
N33	0.0139 (17)	0.0168 (19)	0.0135 (16)	0.0022 (14)	−0.0001 (13)	0.0000 (14)
N29	0.0107 (16)	0.0174 (18)	0.0148 (17)	−0.0017 (14)	0.0021 (13)	0.0022 (14)
N34	0.0182 (18)	0.0146 (18)	0.0191 (18)	0.0034 (14)	−0.0027 (14)	0.0000 (14)
N30	0.0119 (17)	0.0173 (19)	0.0190 (18)	0.0010 (14)	0.0005 (13)	0.0007 (14)
N31	0.0131 (17)	0.0166 (18)	0.0172 (17)	0.0042 (14)	−0.0011 (13)	−0.0036 (13)
N32	0.0182 (19)	0.0171 (19)	0.0147 (18)	0.0028 (15)	−0.0008 (13)	0.0040 (14)
C9	0.014 (2)	0.020 (2)	0.0164 (18)	−0.0002 (18)	0.0033 (16)	0.0046 (16)
C11	0.018 (2)	0.0155 (19)	0.0176 (18)	0.000 (2)	−0.0020 (17)	−0.0015 (15)
C12	0.019 (2)	0.019 (2)	0.0119 (17)	0.0008 (18)	−0.0030 (16)	−0.0010 (14)
C13	0.019 (2)	0.009 (2)	0.025 (2)	−0.0037 (17)	−0.0024 (16)	0.0031 (17)
C14	0.015 (2)	0.015 (2)	0.020 (2)	−0.0006 (16)	0.0009 (16)	0.0007 (17)
C10	0.014 (2)	0.018 (2)	0.018 (2)	−0.0037 (17)	0.0051 (16)	−0.0016 (18)
C1	0.0071 (17)	0.018 (2)	0.0156 (19)	0.0040 (16)	−0.0027 (14)	−0.0018 (17)
C6	0.015 (2)	0.017 (2)	0.0123 (18)	−0.0032 (17)	−0.0016 (15)	0.0022 (16)
N23	0.0146 (17)	0.0209 (19)	0.0181 (17)	0.0018 (15)	0.0014 (13)	−0.0003 (15)
N25	0.0168 (19)	0.026 (2)	0.0197 (18)	−0.0049 (16)	0.0011 (14)	−0.0001 (16)
C7	0.0096 (19)	0.0147 (19)	0.0102 (17)	−0.0033 (15)	−0.0008 (14)	−0.0028 (15)
C2	0.0127 (19)	0.012 (2)	0.0165 (19)	0.0006 (16)	0.0016 (15)	−0.0020 (16)
N27	0.0175 (18)	0.0174 (18)	0.0142 (16)	−0.0010 (14)	0.0007 (13)	−0.0015 (14)
N22	0.0168 (18)	0.021 (2)	0.0172 (17)	−0.0041 (16)	−0.0046 (13)	0.0038 (15)
N21	0.0163 (18)	0.0160 (19)	0.0260 (19)	0.0005 (15)	0.0020 (14)	−0.0015 (16)
C3	0.0098 (18)	0.018 (2)	0.0104 (19)	−0.0046 (16)	−0.0028 (14)	0.0004 (16)
C5	0.0111 (18)	0.013 (2)	0.0102 (18)	0.0025 (16)	0.0028 (14)	0.0007 (16)
C8	0.0139 (19)	0.0147 (19)	0.0102 (16)	−0.0017 (17)	−0.0014 (15)	0.0039 (13)
C4	0.012 (2)	0.017 (2)	0.0132 (19)	−0.0004 (16)	−0.0020 (15)	−0.0021 (16)
O42	0.0262 (17)	0.042 (2)	0.0154 (15)	0.0188 (15)	−0.0059 (13)	−0.0105 (15)
O43	0.0283 (18)	0.0214 (18)	0.0216 (16)	−0.0077 (14)	0.0068 (13)	−0.0017 (14)
N24	0.0221 (19)	0.0154 (17)	0.0185 (16)	−0.0011 (16)	0.0016 (16)	−0.0023 (13)
N26	0.0119 (17)	0.027 (2)	0.0214 (18)	0.0046 (15)	−0.0045 (14)	−0.0077 (16)
N28	0.0152 (18)	0.0132 (18)	0.0176 (17)	0.0004 (14)	−0.0001 (14)	0.0013 (14)
N35	0.0169 (18)	0.0120 (17)	0.0123 (16)	0.0003 (14)	−0.0013 (13)	0.0009 (14)
N36	0.0210 (19)	0.0152 (18)	0.0148 (17)	−0.0015 (15)	−0.0045 (14)	−0.0011 (14)
C15	0.0115 (18)	0.024 (2)	0.023 (2)	0.0011 (16)	0.0026 (17)	−0.002 (2)
C16	0.018 (2)	0.021 (2)	0.019 (2)	0.0060 (17)	0.0020 (16)	0.0003 (18)
O41	0.0107 (14)	0.0178 (14)	0.0150 (13)	−0.0008 (12)	0.0008 (11)	−0.0011 (11)
N37	0.0134 (17)	0.0158 (18)	0.0122 (16)	−0.0037 (14)	0.0018 (12)	−0.0013 (13)
N38	0.0137 (17)	0.0135 (18)	0.0192 (18)	−0.0008 (14)	−0.0020 (13)	0.0029 (14)
C17	0.014 (2)	0.023 (2)	0.015 (2)	−0.0012 (17)	−0.0047 (16)	0.0005 (18)
C18	0.013 (2)	0.017 (2)	0.021 (2)	−0.0020 (17)	−0.0015 (15)	0.0028 (18)

### Geometric parameters (Å, °)

**Table:**

Mo1—C7	2.149 (4)	C12—H12A	0.97
Mo1—C1	2.149 (4)	C13—C14	1.515 (5)
Mo1—C2	2.150 (4)	C13—H13A	0.97
Mo1—C6	2.156 (4)	C13—H13B	0.97
Mo1—C3	2.161 (4)	C14—H14B	0.97
Mo1—C5	2.164 (4)	C14—H14A	0.97
Mo1—C8	2.168 (4)	C10—H10A	0.97
Mo1—C4	2.169 (4)	C10—H10B	0.97
Co2—N27	2.065 (3)	C1—N23	1.152 (4)
Co2—N36	2.099 (3)	C6—N25	1.144 (5)
Co2—N37	2.109 (3)	C7—N27	1.155 (5)
Co2—N35	2.109 (3)	C2—N21	1.155 (5)
Co2—N38	2.113 (3)	N22—C3	1.154 (5)
Co2—O41	2.118 (3)	C5—N28	1.155 (5)
Co1—N32	2.106 (4)	C8—N24	1.158 (4)
Co1—N30	2.122 (3)	C4—N26	1.154 (5)
Co1—N34	2.126 (3)	O42—H42B	0.84 (4)
Co1—N33	2.130 (3)	O42—H42A	0.82 (4)
Co1—N29	2.131 (3)	O43—H43B	0.84 (2)
Co1—N31	2.132 (3)	O43—H43A	0.85 (2)
N33—C11	1.487 (5)	N35—C15	1.475 (5)
N33—H33B	0.90	N35—H35A	0.90
N33—H33A	0.90	N35—H35B	0.90
N29—C9	1.477 (5)	N36—C16	1.485 (5)
N29—H29B	0.90	N36—H36B	0.90
N29—H29A	0.90	N36—H36A	0.90
N34—C12	1.468 (5)	C15—C16	1.526 (6)
N34—H34A	0.90	C15—H15B	0.97
N34—H34B	0.90	C15—H15A	0.97
N30—C10	1.475 (5)	C16—H16A	0.97
N30—H30B	0.90	C16—H16B	0.97
N30—H30A	0.90	O41—H41B	0.85 (2)
N31—C14	1.479 (5)	O41—H41A	0.84 (2)
N31—H31B	0.90	N37—C18	1.479 (5)
N31—H31A	0.90	N37—H37B	0.90
N32—C13	1.476 (5)	N37—H37A	0.90
N32—H32B	0.90	N38—C17	1.475 (5)
N32—H32A	0.90	N38—H38B	0.90
C9—C10	1.500 (6)	N38—H38A	0.90
C9—H9B	0.97	C17—C18	1.524 (5)
C9—H9A	0.97	C17—H17A	0.97
C11—C12	1.507 (5)	C17—H17B	0.97
C11—H11B	0.97	C18—H18A	0.97
C11—H11A	0.97	C18—H18B	0.97
C12—H12B	0.97		
			
C7—Mo1—C1	143.17 (15)	C10—C9—H9B	109.9
C7—Mo1—C2	84.93 (14)	N29—C9—H9A	109.9
C1—Mo1—C2	104.42 (14)	C10—C9—H9A	109.9
C7—Mo1—C6	73.08 (14)	H9B—C9—H9A	108.3
C1—Mo1—C6	79.42 (14)	N33—C11—C12	107.9 (3)
C2—Mo1—C6	143.71 (16)	N33—C11—H11B	110.1
C7—Mo1—C3	142.44 (14)	C12—C11—H11B	110.1
C1—Mo1—C3	73.26 (14)	N33—C11—H11A	110.1
C2—Mo1—C3	73.40 (15)	C12—C11—H11A	110.1
C6—Mo1—C3	139.43 (15)	H11B—C11—H11A	108.4
C7—Mo1—C5	72.19 (14)	N34—C12—C11	108.0 (3)
C1—Mo1—C5	144.18 (15)	N34—C12—H12B	110.1
C2—Mo1—C5	78.89 (14)	C11—C12—H12B	110.1
C6—Mo1—C5	119.18 (13)	N34—C12—H12A	110.1
C3—Mo1—C5	73.74 (14)	C11—C12—H12A	110.1
C7—Mo1—C8	75.50 (13)	H12B—C12—H12A	108.4
C1—Mo1—C8	74.01 (14)	N32—C13—C14	109.2 (3)
C2—Mo1—C8	71.78 (15)	N32—C13—H13A	109.8
C6—Mo1—C8	74.91 (15)	C14—C13—H13A	109.8
C3—Mo1—C8	123.39 (14)	N32—C13—H13B	109.8
C5—Mo1—C8	137.76 (14)	C14—C13—H13B	109.8
C7—Mo1—C4	109.20 (14)	H13A—C13—H13B	108.3
C1—Mo1—C4	83.56 (14)	N31—C14—C13	109.5 (3)
C2—Mo1—C4	145.00 (15)	N31—C14—H14B	109.8
C6—Mo1—C4	70.90 (15)	C13—C14—H14B	109.8
C3—Mo1—C4	76.66 (15)	N31—C14—H14A	109.8
C5—Mo1—C4	75.64 (14)	C13—C14—H14A	109.8
C8—Mo1—C4	141.90 (15)	H14B—C14—H14A	108.2
N27—Co2—N36	87.94 (13)	N30—C10—C9	109.2 (3)
N27—Co2—N37	173.08 (13)	N30—C10—H10A	109.8
N36—Co2—N37	93.78 (13)	C9—C10—H10A	109.8
N27—Co2—N35	91.53 (13)	N30—C10—H10B	109.8
N36—Co2—N35	82.67 (12)	C9—C10—H10B	109.8
N37—Co2—N35	95.34 (12)	H10A—C10—H10B	108.3
N27—Co2—N38	91.17 (13)	N23—C1—Mo1	177.8 (4)
N36—Co2—N38	96.88 (13)	N25—C6—Mo1	179.3 (4)
N37—Co2—N38	81.97 (12)	N27—C7—Mo1	174.6 (3)
N35—Co2—N38	177.24 (13)	N21—C2—Mo1	177.3 (4)
N27—Co2—O41	92.32 (12)	C7—N27—Co2	159.1 (3)
N36—Co2—O41	168.90 (12)	N22—C3—Mo1	178.2 (3)
N37—Co2—O41	87.28 (11)	N28—C5—Mo1	177.7 (3)
N35—Co2—O41	86.23 (12)	N24—C8—Mo1	177.9 (3)
N38—Co2—O41	94.21 (12)	N26—C4—Mo1	178.4 (3)
N32—Co1—N30	169.59 (12)	H42B—O42—H42A	103 (3)
N32—Co1—N34	94.94 (13)	H43B—O43—H43A	110 (3)
N30—Co1—N34	94.64 (13)	C15—N35—Co2	108.3 (2)
N32—Co1—N33	94.55 (13)	C15—N35—H35A	110
N30—Co1—N33	90.88 (13)	Co2—N35—H35A	110
N34—Co1—N33	81.55 (12)	C15—N35—H35B	110
N32—Co1—N29	89.02 (12)	Co2—N35—H35B	110
N30—Co1—N29	81.55 (12)	H35A—N35—H35B	108.4
N34—Co1—N29	175.62 (13)	C16—N36—Co2	108.2 (2)
N33—Co1—N29	96.29 (12)	C16—N36—H36B	110.1
N32—Co1—N31	81.96 (13)	Co2—N36—H36B	110.1
N30—Co1—N31	93.99 (13)	C16—N36—H36A	110.1
N34—Co1—N31	89.88 (13)	Co2—N36—H36A	110.1
N33—Co1—N31	170.46 (12)	H36B—N36—H36A	108.4
N29—Co1—N31	92.54 (13)	N35—C15—C16	108.2 (3)
C11—N33—Co1	107.3 (2)	N35—C15—H15B	110.1
C11—N33—H33B	110.3	C16—C15—H15B	110.1
Co1—N33—H33B	110.3	N35—C15—H15A	110.1
C11—N33—H33A	110.3	C16—C15—H15A	110.1
Co1—N33—H33A	110.3	H15B—C15—H15A	108.4
H33B—N33—H33A	108.5	N36—C16—C15	108.5 (3)
C9—N29—Co1	108.6 (2)	N36—C16—H16A	110
C9—N29—H29B	110	C15—C16—H16A	110
Co1—N29—H29B	110	N36—C16—H16B	110
C9—N29—H29A	110	C15—C16—H16B	110
Co1—N29—H29A	110	H16A—C16—H16B	108.4
H29B—N29—H29A	108.4	Co2—O41—H41B	111 (3)
C12—N34—Co1	108.3 (2)	Co2—O41—H41A	108 (3)
C12—N34—H34A	110	H41B—O41—H41A	112 (3)
Co1—N34—H34A	110	C18—N37—Co2	107.5 (2)
C12—N34—H34B	110	C18—N37—H37B	110.2
Co1—N34—H34B	110	Co2—N37—H37B	110.2
H34A—N34—H34B	108.4	C18—N37—H37A	110.2
C10—N30—Co1	108.3 (2)	Co2—N37—H37A	110.2
C10—N30—H30B	110	H37B—N37—H37A	108.5
Co1—N30—H30B	110	C17—N38—Co2	109.3 (2)
C10—N30—H30A	110	C17—N38—H38B	109.8
Co1—N30—H30A	110	Co2—N38—H38B	109.8
H30B—N30—H30A	108.4	C17—N38—H38A	109.8
C14—N31—Co1	109.5 (2)	Co2—N38—H38A	109.8
C14—N31—H31B	109.8	H38B—N38—H38A	108.3
Co1—N31—H31B	109.8	N38—C17—C18	108.2 (3)
C14—N31—H31A	109.8	N38—C17—H17A	110.1
Co1—N31—H31A	109.8	C18—C17—H17A	110.1
H31B—N31—H31A	108.2	N38—C17—H17B	110.1
C13—N32—Co1	107.5 (2)	C18—C17—H17B	110.1
C13—N32—H32B	110.2	H17A—C17—H17B	108.4
Co1—N32—H32B	110.2	N37—C18—C17	107.8 (3)
C13—N32—H32A	110.2	N37—C18—H18A	110.1
Co1—N32—H32A	110.2	C17—C18—H18A	110.1
H32B—N32—H32A	108.5	N37—C18—H18B	110.1
N29—C9—C10	108.9 (3)	C17—C18—H18B	110.1
N29—C9—H9B	109.9	H18A—C18—H18B	108.5

### Hydrogen-bond geometry (Å, °)

**Table:**

*D*—H···*A*	*D*—H	H···*A*	*D*···*A*	*D*—H···*A*
N29—H29A···N22^i^	0.90	2.59	3.313 (5)	138
N29—H29B···N24^ii^	0.90	2.45	3.243 (5)	147
N30—H30A···N26^iii^	0.90	2.39	3.121 (5)	139
N31—H31B···N24^ii^	0.90	2.22	3.079 (4)	160
N32—H32A···N22^i^	0.90	2.4	3.163 (5)	143
N32—H32B···N26^iv^	0.90	2.16	3.037 (5)	164
N33—H33B···O41^v^	0.90	2.51	3.233 (4)	138
N34—H34A···N28^iv^	0.90	2.53	3.213 (5)	133
N34—H34B···N26^iii^	0.90	2.53	3.408 (5)	164
N36—H36A···N22^vi^	0.90	2.31	3.195 (4)	166
N36—H36B···N23^vi^	0.90	2.56	3.151 (5)	124
N37—H37A···N22^vi^	0.90	2.22	3.103 (5)	167
N37—H37B···N21^vii^	0.90	2.17	3.035 (5)	162
N38—H38A···N25^viii^	0.90	2.49	3.282 (5)	148
N38—H38B···O42^vi^	0.90	2.47	3.350 (5)	164
O41—H41A···N25^viii^	0.84 (2)	1.94 (2)	2.764 (4)	170 (4)
O41—H41B···O43^vi^	0.85 (2)	1.89 (2)	2.724 (4)	168 (4)
O42—H42A···N28^v^	0.82 (4)	2.10 (4)	2.924 (4)	173 (4)
O42—H42B···N23	0.84 (4)	1.96 (4)	2.799 (4)	174 (4)
O43—H43A···N24^v^	0.85 (2)	2.15 (2)	2.995 (4)	174 (4)
O43—H43B···O42^ix^	0.84 (3)	1.95 (2)	2.778 (4)	168 (4)
C10—H10B···N23^iii^	0.97	2.53	3.328 (5)	139
C11—H11B···O42^ix^	0.97	2.59	3.430 (5)	145

Symmetry codes: (i) −*x*, *y*−1/2, −*z*+3/2; (ii) *x*, *y*−1, *z*; (iii) −*x*+1, *y*−1/2, −*z*+3/2; (iv) −*x*+1/2, −*y*, *z*+1/2; (v) −*x*+1/2, −*y*+1, *z*+1/2; (vi) −*x*+1/2, −*y*+1, *z*−1/2; (vii) *x*+1/2, −*y*+3/2, −*z*+1; (viii) *x*−1/2, −*y*+3/2, −*z*+1; (ix) *x*−1/2, −*y*+1/2, −*z*+2.
